# Basic considerations on the practical method for predicting sound insulation performance of a single-leaf window

**DOI:** 10.14324/111.444/ucloe.000018

**Published:** 2021-05-07

**Authors:** Yohei Tsukamoto, Kimihiro Sakagami, Takeshi Okuzono, Yoshihiro Tomikawa

**Affiliations:** 1 YKK AP Inc., YKK AP R&D Centre, 1 Ogyu, Kurobe, Toyama 938-8612, Japan; 2 Environmental Acoustists Laboratory, Graduate School of Engineering, Kobe University, 1-1 Rokkodai, Nada, Kobe, Hyogo 657-8501, Japan

**Keywords:** architectural acoustics, mass law, measurement, single glazing, sound insulation, window

## Abstract

As a basic study of a practical method for predicting the sound insulation performance of windows, this report presents a study of the sound reduction index of windows with single glazing, below a critical frequency. First, the results calculated by an existing theory for a single plate for the sound reduction indices are compared with measured results of actual windows to assess the theory’s applicability for evaluating the sound insulation performance of windows. Then, a regression analysis is employed to measure the results of a certain number of actual windows to explore a further development of a more practical prediction. The following findings were obtained: (1) Sound reduction indices of actual fixed windows are predictable using Sewell’s transmission theory for a single plate. However, sound reduction indices of openable windows, especially those of sliding windows, are strongly affected by gaps in the window frame. Therefore, predicting sound reduction indices of all windows accurately is difficult if using only one theory. (2) The frequency slope of the window reduction index is much lower than that of the mass law. Regression analyses indicate that the frequency slope of the reduction index of all examined windows is 3.0 dB per octave, on average.

## Introduction

Indoor environmental quality (IEQ) is of paramount importance in houses, offices and most types of buildings. Among various environmental factors, a quiet indoor acoustic environment is necessary both in living and working places, to ensure good quality conditions for human activities within those spaces, and this depends on, for example, the sound insulation performance of exterior walls of buildings [1]. The indoor acoustic environment depends mainly on the sound insulation performance of the windows because these are the weakest components of exterior walls in many cases [2]. In acoustic environment design, predicting the sound insulation performance of windows is crucially important. Many points of consideration exist in window design, including obtaining natural ventilation, day lighting and the view from a building. In effect, these functions often share a trade-off relation with sound insulation, which has led to the development of a wide variety of window types, with sound insulation performance depending on the type. In addition to conventional ventilation windows, various types of natural ventilation windows such as plenum windows have recently become a focus of design. The sound insulation performance of windows including the natural ventilation type was reviewed by Tang [3] and they concluded that a plenum window is an interesting alternative that is worth considering. This concept led to another approach regarding the performance of windows, especially natural-ventilating windows, and an evaluation of them for perceptual factors using the indoor soundscape approach [4]. These new trends will become more important in the future, but for now the physical assessment of the sound insulation performance of conventional ventilation windows is still necessary, as has been the general approach to date. Therefore, it is still important to develop a practical prediction method for the sound reduction index that is applicable for windows of different types because the fundamental sound insulation performance of the window is evaluated in closed conditions.

Although the mass law of a single plate gives a slope of 6 dB per octave [1], reduction indices of single glazing in an actual window frame usually indicate different values. It is therefore considered that the mass law is not applicable to an actual size of windows in usual buildings. Some other effects that are not observed in walls are unique to windows themselves, such as air gaps between sashes and frames or degrees of fixation depending on the window type. Additionally, the windowpane size should be a consideration when predicting the sound insulation of windows. Therefore, a simple mass law-based discussion would not be sufficient for the present purpose: we must start with a theory that can accommodate the panel size effect.

For this purpose, the theory developed by Sewell [5] is regarded as promising. Sewell developed an expression for reduction indices of finite single plates considering the effects of plate size, although the theory is limited to below coincidence frequency. Although Sewell’s theory is not aimed at finite-sized sashed windows, it considers differences in radiation efficiencies depending on the plate size. Quirt studied sound transmission characteristics experimentally with modelled windows [6], and found good correlation with the experimental results. However his model was considerably simplified, it is still not very clear how well the theory can describe the sound insulation performance of actual windows. Regarding the gap, some cases exist in which the effects are studied using numerical analysis [7]. However, no practical formula to predict or estimate window frame effects on the sound insulation performance of a window has been proposed. Iwase and colleagues [8–11] attempted to evaluate gap effects experimentally using a modelled reverberation chamber. However, they did not present definite results that are applicable to practical cases. As there are fewer studies which show the data of a certain number of actual windows, the general characteristics of sound insulation performance of an actual window are less known. Therefore, establishing a practical method to predict the sound insulation performance of an actual window with a simple expression is of paramount importance.

The goal of this project was to establish a practical method to show the sound insulation performance of a window. As a basic study, the sound reduction index of a single-leaf window below its critical frequency is discussed here. First, we applied Sewell’s theory for a single plate by comparing the calculated and measured results of reduction indices of actual windows. The results of the comparison showed the applicability of Sewell’s theory. Second, as another alternative for obtaining a practical solution, we tried to apply regression analyses to our measured data of reduction indices of windows. For this purpose, we used a set of measured data of 83 windows of three types; regression lines for windows of each type were obtained to confirm the slope of the frequency characteristics of the reduction index. It should be noted that this study is limited to conventional ventilation windows in closed conditions because the basic sound insulation performance of windows is mainly evaluated in closed conditions in Japan.

## Prediction using Sewell’s theory

### Fundamental principles of theoretical prediction of reduction index

According to the well-known mass law, the field-incidence-averaged sound reduction index *R_m_* is given by the following expression [12].

(1) $$R_{m}=10\log{\left(\frac{\omega m}{2\rho c}\right)}^{2}-5$$

where *ω* stands for angular frequency, *m* (kg/m^2^) denotes the plate surface density, *ρ* (kg/m^3^) expresses air density, and *c* (m/s) signifies the sound velocity in air. As the mass law [Eq. (1)] is based on the assumption of an infinite plate in piston-like motion to a plane wave, it agrees with the measured result of a large homogeneous wall. Basically, the mass law in its original form is given for a normal incidence of a plane wave, but Eq. (1) is modified to adapt it for use in a diffuse sound field case by adding a correction factor of 5 [12].

However, for small plates, it cannot agree with the measured results. In most cases, *R_m_* by Eq. (1) does not agree with measured results of a window’s reduction index. Sewell derived a formula for evaluating a reduction index for a finite plate with sound-induced vibration that is applicable below the coincidence frequencies as follows;

(2) $$R_{s}=10\log\left(\frac{1}{\tau_{s}}\right)$$

(3) $$\tau_{s}={\left\{\left(\frac{\omega m}{2\rho c}\right)\left[1-\frac{\omega^{2}}{\omega_{c}^{2}}\right]\right\}}^{-2}\left[\ln\left(k\sqrt{F}\right)+0.160-U(\Lambda )+\frac{1}{4\pi k^{2}F}\right]$$

(4) $$U(\Lambda )=-0.804-\left(\frac{1}{2}+\frac{\Lambda }{\pi }\right)\ln\Lambda +\frac{5\Lambda }{2\pi }+\sum_{n=1}^{\infty }\frac{{\left(-1\right)}^{n-1}\Lambda^{2n+1}}{2\pi n(n+1){\left(2n+1\right)}^{2}}$$

In these equations, *k* = *ω*/*c* expresses the wave number, *F* (m^2^) denotes the sample area, and *Λ* represents the ratio of the shorter side length to the longer side length of the sample.

## Measurement data

The data used in this study are existing test data accumulated in a manufacturer’s database, in which all data were taken from the product of one manufacturer. All measurements were performed according to JIS A 1416 [13], which is compatible with ISO 10140-2 [14]. Although the configurations of the measurement samples are not necessarily the same, the sizes of the sample windows are based on typical use. By using the measured data of several different product samples in every window type and every glass thickness, as much data as possible was collected in each category. We considered that the multiple measured data reduce the uncertainty and improves the reliability of the measurement. All measurements were taken in reverberation chambers which have coupled reverberant rooms: the source room had a volume of 492.8 m^3^ while the receiving room had a volume of 264.5 m^3^. Reduction indices in the 1/3 octave bands were calculated from 100 to 5000 Hz according to the standard.

### Comparing theoretical results and measurement results

**Figure 1:**
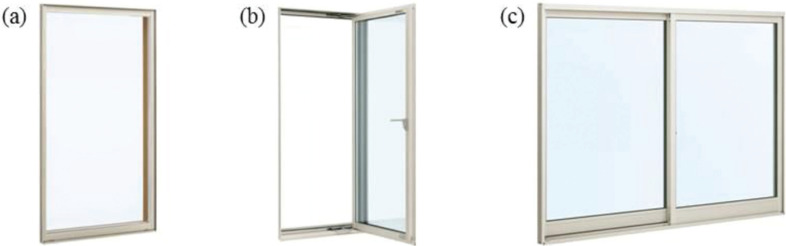
Sketches of the measured window types. (a), (b) and (c) are a fixed window, a projected window and a sliding window, respectively.

The calculated results using Sewell’s theory for the reduction index of a plate were compared with the measured results of windows of different types. Its applicability for predicting the sound insulation performance of windows was evaluated. The sketches of the measured windows of three types (fixed, projected, sliding) are shown in Fig. 1. The measurements of each window consist of the glass and the frame. The fixed window is not openable. The projected window and the sliding window are defined here as follows: the projected window is an openable window where the top and the bottom of the frame are connected with a sash using friction stay, and the sliding window refers to a window composed of two sashes moving horizontally. The three windows are divided according to the thickness of the glass. The single glazing considered here is commonly used float glass, with 5, 6 and 8 mm thicknesses. Regarding the window size, the samples are organised into three categories according to the approximate sizes: window size *F* (m^2^) is within 15% of the represented value shown in the figures. Up to three measured results are plotted in each graph. It is noteworthy that the data used for this comparison include particular trends of windows: thicker glazing tends to be used for higher-specification windows intended for use in high-rise buildings. Because these data are obtained from actual commercial windows, this tendency is reflected in this study.

#### Fixed window

**Figure 2:**
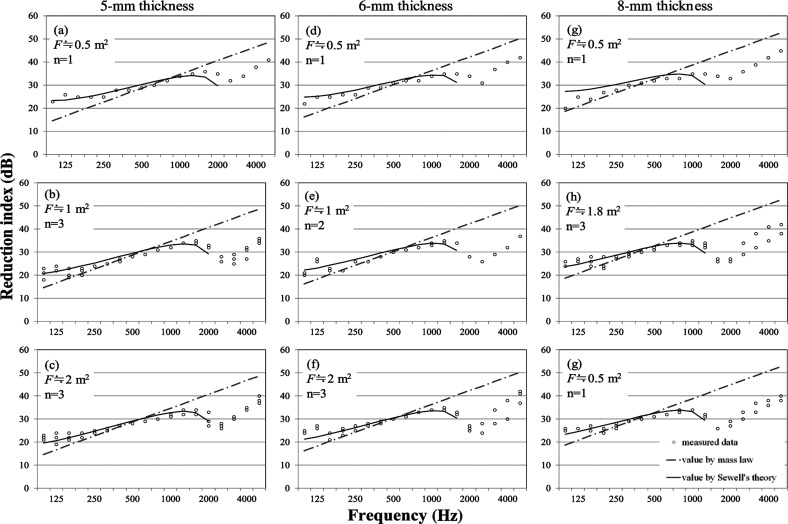
Reduction indices of fixed window with theories. Solid lines and dash–dotted lines, respectively, represent values using Sewell’s theory and values using the mass law. Plots show measured data of windows in the 1/3 octave. ‘F’ and ‘n’ represent the window area and the number of the samples, respectively.

Figure 2 portrays the measured results of reduction indices of fixed windows with the corresponding results obtained using Sewell’s formula [Eqs (2–4)]. From the left side, the results obtained for 5-, 6- and 8-mm glass thicknesses are shown. The examined fixed windows with sizes of 0.5–2.0 m^2^ are shown. Usually the size of fixed windows ranges are almost the same as those of projected windows, however, in this study somewhat smaller fixed windows were included. The sizes are classified into three levels for each thickness in Fig. 2. Moreover, the field incidence mass law values for the same surface density are presented in the same figure for a reference. According to Fig. 2, the measured values have markedly lower slopes than the mass law: at 125 Hz, the measured values are greater than the mass law, but above 1000 Hz, they are consistently smaller than the mass law in all conditions. Sewell’s calculated values show good agreement with the measured results of the windows, especially at low frequencies. Therefore, Sewell’s theory for a plate appears to be applicable for predicting the reduction index of fixed windows. However, there is a following mismatching trend. The calculated results by Sewell’s theory show greater values for smaller windows. However the experimentally obtained results, regardless of size, show similar reduction indices to each other. They are not regarded as depending on the window size.

#### Projected window

**Figure 3:**
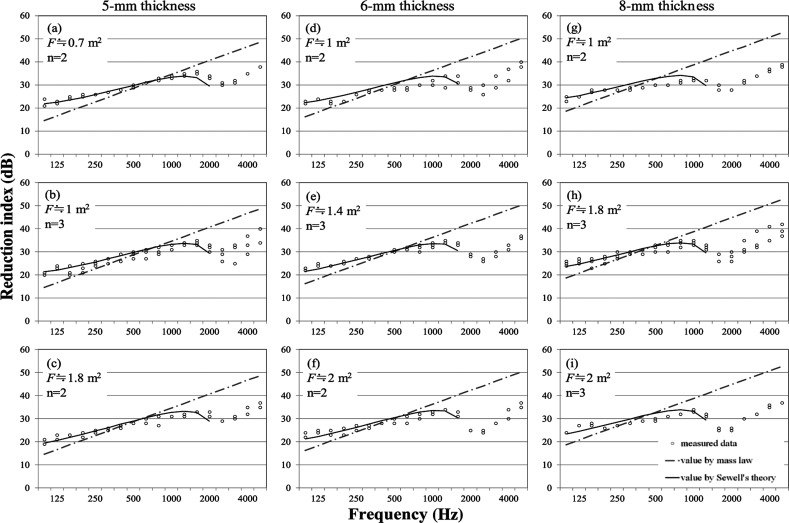
Reduction indices of the projected window with theories. Solid lines and dash–dotted lines, respectively, represent values using Sewell’s theory and values using the mass law. Plots show measured data of windows in the 1/3 octave. ‘F’ and ‘n’ represent the window area and the number of the samples, respectively.

Measured results for projected windows with the corresponding calculated results are presented in Fig. 3, as described in the fixed window’s section. The examined projected windows with sizes of 0.7–2.0 m^2^ are classified into three levels according to their respective thicknesses. The measurement results are expected to vary by the type of window, because openable windows have a more complex construction than fixed windows. But according to Fig. 3, a major trend of the results of projected windows is similar to the trend found for fixed windows. The measured values agree with Sewell’s calculated values rather than the mass law. As described for fixed windows, the measured results are not considered to depend on the window size. However, at around 500–1000 Hz, some downward deviation from Sewell’s curve is apparent. This downward deviation can be attributed to the effects of gaps and degrees of fixation of the window frame.

#### Sliding window

**Figure 4:**
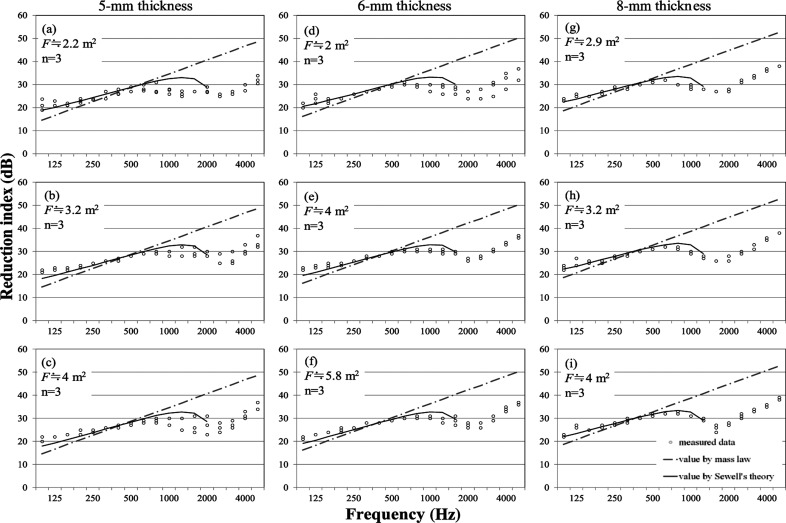
Reduction indices of sliding window with theories. Solid lines and dash–dotted lines, respectively, represent values using Sewell’s theory and values using the mass law. Plots show measured data of windows in the 1/3 octave. ‘F’ and ‘n’ represent the window area and the number of the samples, respectively.

Measured results obtained for sliding windows with the corresponding calculated results are depicted in Fig. 4 as described in earlier sections. The examined sliding windows with sizes of 2.0–5.8 m^2^ are shown, with sizes classified into three levels for each thickness in Fig. 4. According to Fig. 4, greater variation exists between samples under the same conditions than in fixed and projected windows. Especially at around 1000 Hz, many samples show much lower values than Sewell’s curve. One of the main causes of this deterioration is attributable to the sash gaps because the sliding window tends to create greater gaps structurally than in windows of other types. The values of windows with 8-mm thickness glazing do not seem to decrease at middle frequencies, probably because sashes and frames with such thick glass are designed for high airtightness. Regarding the size, the sample sizes of sliding windows are bigger than the other types because the sliding windows consist of two panes with two sashes. Sewell’s curves of samples with large sizes are strongly affected by sizes showing lower values than the results measured at low frequencies. Consequently, Sewell’s theory tends to present a low value for large windows. Thus, Sewell’s theory cannot apply for predicting the reduction indices of sliding windows.

#### Discussion

**Figure 5:**
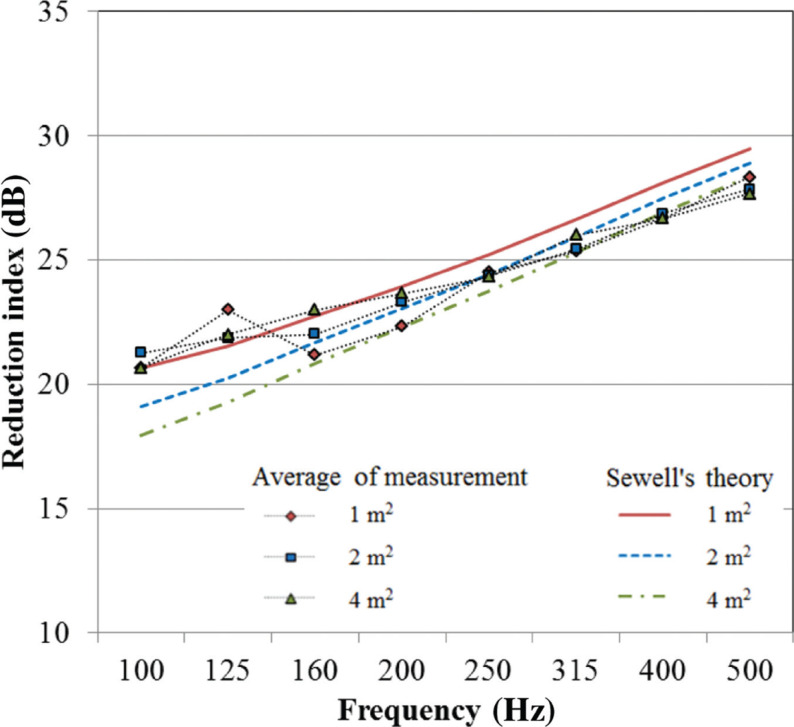
Reduction indices of windows with 5-mm thick glazing. Comparison of experimental and theoretical size contributions.

For fixed windows with single glazing, Sewell’s prediction theory shows good agreement with the measured values of the reduction index. However, despite single glazing, in the case of openable windows and especially sliding windows, Sewell’s curve disagrees with the measured values at the middle frequencies. That result is attributable to the effects of window frames including gaps and degrees of fixation. However, the decrement cannot be predicted. The theory can be interpreted as it describes the sound insulation performance in the ideal condition, for example, perfectly airtight without any gaps, etc. With respect to the effect of the size at low frequencies, Fig. 5 shows the average measurement reduction indices for windows of all types with 5-mm thick glazing used in this section with the corresponding theory by sizes. Sewell’s curves exhibit a trend by which larger plates show a lower reduction index. However, the measured results of all sizes of windows show that similar values constantly exceed 20 dB at low frequencies, unlike Sewell’s theoretical values. This tendency is noteworthy because, in the case of the sound insulation of walls, the reduction index is well known to agree with Sewell’s predictions in many cases. Although the reason for this discrepancy remains an unresolved problem, the presented results demonstrate that, for the practical prediction of the sound reduction index of windows, it is not particularly important to include the window pane size effect.

Consequently, for the prediction of the sound insulation performance of windows including sliding windows, Sewell’s theory can be informative. However, it is impossible to accomplish by depending solely on Sewell’s theory. It is necessary that the prediction method of the reduction indices of windows be distinguished at least by the window type. Additionally, from the results in this section, shared trends are visible that show that reduction indices of windows have linear frequency characteristics with constant slopes in the frequency range that are not affected by the coincidence effect. As a shorter method to express the characteristics of sound insulation of a window, a regression analysis is performed in next section.

## Prediction by regression analysis

### Analysis procedure

The statistical prediction method of the sound reduction index for a single-glazed window is studied. A number of measured samples are divided based on the window type and glass thickness. Because the window size effect was regarded as unimportant based on earlier discussions, this analysis is not divided by size. Windows are categorised into three types (fixed, projected, sliding), with glass of three thicknesses (5, 6 and 8 mm); up to ten measured results of reduction indices falling into the categories are shown, respectively. The data used for the analyses are taken from the same database as described in the section Measurement Data. Linear regression is applied to the average of ten samples in the range below half of the critical frequency to observe the frequency characteristics without a coincidence effect. This is because the coincidence effect appears above half of the critical frequency. Some conditions did not get ten samples, as shown by the number in each graph. In addition, measured data of the reduction indices of single glazing without window frames [15] and the field incidence mass law are shown in the same figure for reference. From the regression results, the slopes of the reduction indices of windows of different types are calculated.

### Analysis result

**Figure 6:**
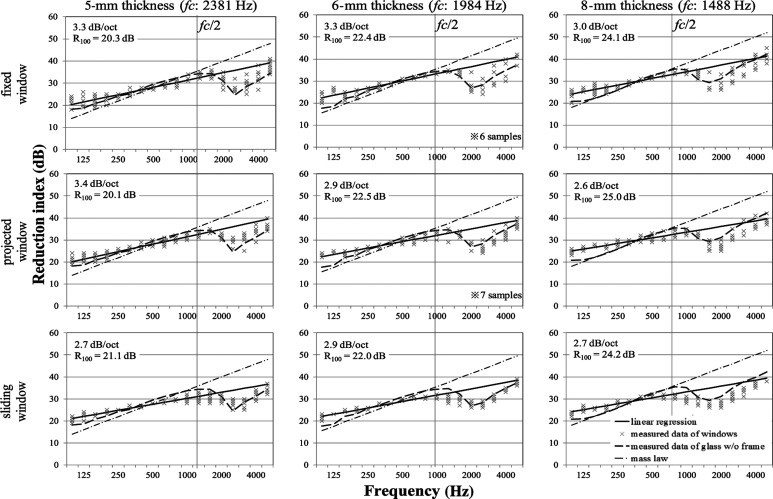
Reduction indices of ten samples for each condition. Solid lines show the results of linear regression for an average of ten samples in the range 100 Hz to *fc*/2. Dashed lines and dash–dotted lines, respectively, represent measured values of a single pane of glass without a frame and calculated values using the mass law.

The analysis results are displayed in Fig. 6 for nine categories: from left to right, the results of 5-, 6- and 8-mm glass thicknesses are shown; from top to bottom the results obtained for fixed, projected, and sliding window types are shown. The plots show data of ten sample reduction indices. The solid line shows the linear regression result. It is noted that the line above half of the critical frequency is the extrapolation. Although the ten samples in each graph in Fig. 6 have the same glazing and same type of frame, all conditions show variations. Therefore, despite using the same glass, the sound insulation performance of a window varies because of the structural differences or other window features.

Single-pane glass without a frame shows a slope of about 6 dB/oct below the critical frequency as the general theory of mass law shows. However, no case exists in which the reduction index of a window agrees with the slope of the mass law. These behaviours below their critical frequencies tend to show linear increases with a characteristic slope that differs from the mass law. Figure 6 presents slope values for each condition. The slope values for fixed windows show a 3–3.3 dB/oct increase; those for sliding windows show a 2.7–2.9 dB/oct increase. The cause of the lower slopes of sliding windows is the marked deterioration of the reduction index of sliding windows at around 1000 Hz. This finding is attributed to the effects of gaps. The slope for projected window shows wide variation: 2.6–3.4 dB/oct. Because the projected window has an openable sash, individual differences exist. Presumably, there are variable states from almost fixed windows to windows with a wide gap such as a sliding window. The average slope of all windows used in the present exam is 3.0 dB/oct. Furthermore, for regression, *R*_100_ is presented as a cue for the height value. *R*_100_ in Fig. 6 shows a value of the regression at 100 Hz that is readable from the graph. The thicker the glass that is used, the higher a value of *R*_100_ that is shown.

### Application of regression

**Figure 7:**
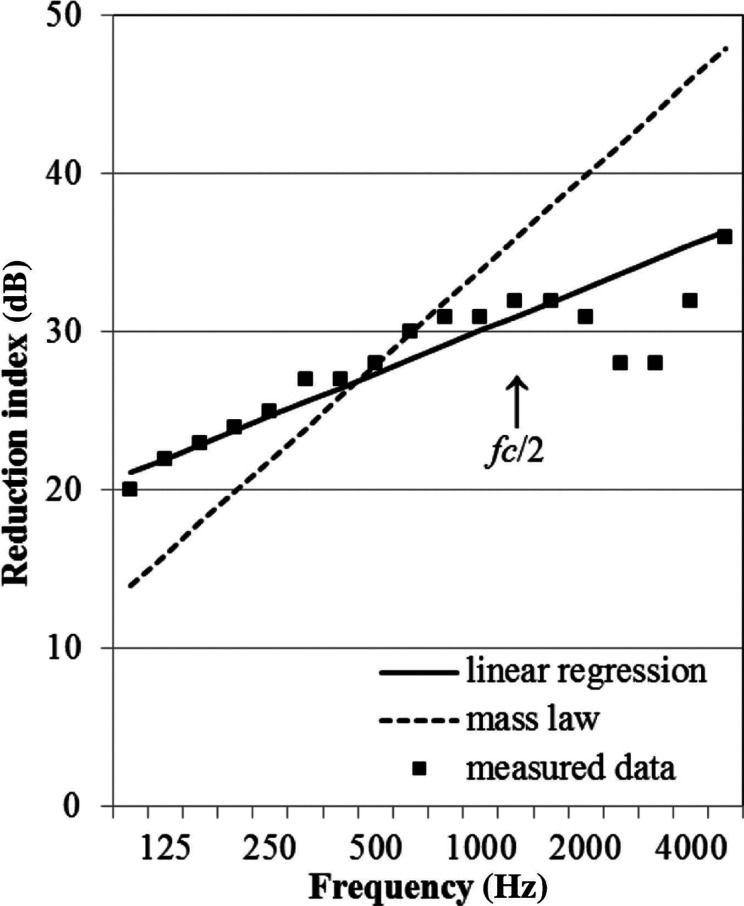
Reduction index of a sliding window. The solid line represents the result of the regression analysis. The dashed line represents values using the mass law.

As ideas that elucidate the practical prediction method of the reduction index of a window, regression equations from the preceding section are explained. This regression application is also useful to confirm the slope of the frequency characteristics of the reduction index of windows. As an example, the case of a sliding window with 5-mm thick glass is demonstrated as follows. According to Fig. 6, in the case of a sliding window with 5-mm thick glass, the regression equation is a straight line that increases by 0.9 dB every 1/3 octaves. Expressed as an equation, it becomes *R* = 9 log *f* + 3.1 (dB). This result is depicted in Fig. 7. The solid line shows this regression equation; the dashed line shows the field incidence mass law for reference; the plots demonstrate other measured data of the window in the same category. In this case, the regression line shows good agreement with measured data: the maximum error is −1.8 dB at 800 Hz. The measured value exceeds the regression line at middle frequencies, probably because this window has good airtightness. The value would be slightly lower than the regression line in these frequencies if a window with weak airtightness was measured. In any case, the measured values of the reduction indices of sliding windows would not deviate from the regression line. Consequently, this can be inferred as the simplest method of practical prediction of sound insulation performance for a window. This regression line is regarded as an important cue for practical predictions. However, the utility of the method is limited below the critical frequency. Further study must be conducted on the prediction of the reduction indices of windows above the critical frequency.

## Conclusions

A basic study was conducted to explore methods for the practical prediction of the sound insulation performance of windows. First, Sewell’s theory for walls, which incorporates panel size effects, was applied to windows. The results were compared with the measured results of window reduction indices. Next, a regression analysis was performed from the measured results of ten samples for each window type and each glass thickness. Consequently, the following findings were obtained:

1. Sewell’s theory showed good agreement with measured results obtained for fixed windows with single glazing. Sewell’s theory for a plate appears to be applicable for predicting the reduction index of a fixed window. However, sound reduction indices of openable type windows, especially of sliding windows, were affected remarkably by window frame gaps. Consequently, accurately predicting sound reduction indices of all windows is difficult when using Sewell’s formula alone. The results also clarified that Sewell’s formula overestimates the effect of window pane size.
2. The frequency slope of windows is much lower than the general theory of mass law. There are characteristics of slopes below the critical frequencies for windows of different types. The frequency slope values for fixed windows are 3–3.3 dB/oct. Those for sliding windows show 2.7–2.9 dB/oct. Regression analysis shows that the reduction index slope with the frequency of all windows used is an average 3.0 dB/oct.

As described here, important cues were obtained for the practical prediction of the sound insulation performance of a window. However, the range of the problem remains limited to single glazing below the critical frequency. Sound reduction indices of multiple glazing or above the coincidence frequency are also important matters that will be the subject of future work.

## Data Availability

The datasets generated during and/or analysed during the current study are available from the corresponding author on reasonable request.
